# African Program for Onchocerciasis Control 1995–2010: Impact of Annual Ivermectin Mass Treatment on Off-Target Infectious Diseases

**DOI:** 10.1371/journal.pntd.0004051

**Published:** 2015-09-24

**Authors:** Stanimira P. Krotneva, Luc E. Coffeng, Mounkaila Noma, Honorat G. M. Zouré, Lalle Bakoné, Uche V. Amazigo, Sake J. de Vlas, Wilma A. Stolk

**Affiliations:** 1 Department of Public Health, Erasmus MC, University Medical Center Rotterdam, Rotterdam, The Netherlands; 2 African Programme for Onchocerciasis Control, Ouagadougou, Burkina Faso; 3 Independent consultant, Enugu, Nigeria; Swiss Tropical and Public Health Institute, SWITZERLAND

## Abstract

Since its initiation in 1995, the African Program for Onchocerciasis Control (APOC) has had a substantial impact on the prevalence and burden of onchocerciasis through annual ivermectin mass treatment. Ivermectin is a broad-spectrum anti-parasitic agent that also has an impact on other co-endemic parasitic infections. In this study, we roughly assessed the additional impact of APOC activities on the burden of the most important off-target infections: soil-transmitted helminthiases (STH; ascariasis, trichuriasis, hookworm, and strongyloidiasis), lymphatic filariasis (LF), and scabies. Based on a literature review, we formulated assumptions about the impact of ivermectin treatment on the disease burden of these off-target infections. Using data on the number of ivermectin treatments in APOC regions and the latest estimates of the burden of disease, we then calculated the impact of APOC activities on off-target infections in terms of disability-adjusted life years (DALYs) averted. We conservatively estimated that between 1995 and 2010, annual ivermectin mass treatment has cumulatively averted about 500 thousand DALYs from co-endemic STH infections, LF, and scabies. This impact comprised approximately an additional 5.5% relative to the total burden averted from onchocerciasis (8.9 million DALYs) and indicates that the overall cost-effectiveness of APOC is even higher than previously reported.

## Author Summary

Onchocerciasis, or river blindness, is an infectious disease caused by the worm *Onchocerca volvulus*, which is transmitted between humans through the bites of blackflies and causes deforming skin disease, itch, and vision loss. The African Programme for Onchocerciasis Control (APOC) aims to control morbidity due to onchocerciasis by implementing mass drug administration (MDA) with ivermectin in endemic areas, targeting the whole population except for children under five and pregnant women. Aside from its effect on onchocerciasis, ivermectin also affects other parasitic infections such as lymphatic filariasis, intestinal worm infections, and scabies, which are all significantly co-endemic in areas covered by APOC. In this paper, the researchers roughly estimate the health impact of ivermectin MDA on off-target infections based on the number of dispensed treatments up to 2010, published estimates of the disease burden of off-target infections, and the expected effect of ivermectin treatment on the burden of these infections (based on literature review). This off-target health impact of APOC constitutes about 500 thousand years worth of healthy years of life (an additional 5.5% on top of the impact of APOC on the burden of onchocerciasis) and indicates that the cost-effectiveness of APOC is even higher than previously estimated.

## Introduction

The African Program for Onchocerciasis Control (APOC) is an international program aimed at controlling the disease burden of human onchocerciasis (river blindness) in sub-Saharan Africa (SSA), and elimination of infection where possible, using mass drug treatment (MDA) [1,2]. Since its launch in 1995, APOC and partnering beneficiary countries have scaled up their control activities geographically to at least cover all meso- and hyperendemic areas, averting 8.9 million disability-adjusted life years (DALYs) through 2010, and eventually aiming to treat over 90 million people annually in 16 African countries by 2015, protecting a population at risk of onchocerciasis of 118 million [3,4]. The drug used for mass treatment of onchocerciasis, ivermectin, is distributed and administered in a single dose of 150–200 μg/kg of body weight annually. Chronically ill people, pregnant (or lactating) women, and children under five are excluded from treatment with ivermectin [1].

Ivermectin is known to be effective against various infectious diseases other than onchocerciasis, the most important being soil-transmitted helminth (STH) infections, lymphatic filariasis (LF), and epidermal parasitic skin diseases (EPSDs) such as scabies [5–11]. In APOC countries, the prevalence of STH infections in school-age children ranges between 20% and 50% [12]. LF is endemic in all APOC countries with an estimated overall prevalence of 6–9% [10], and local prevalences typically ranging between 0–40% [13]. Despite the lack of comprehensive epidemiological data, it is known that EPSDs are prevalent across SSA and that the associated morbidity is significant in regions of high poverty [9,14,15]. Together, these infections are responsible for a considerable burden of disease [14,15]. Therefore, annual mass treatment with ivermectin is expected to have an additional health impact by averting part of the burden related to these off-target infections [8,16–19]. Although these additional beneficial effects of ivermectin are being used to sensitise communities to participate in MDA, up till now, the off-target health impact has not been quantified and its importance remains unknown.

In this study, we quantified the health impact of APOC activities through 2010 on the burden of STH (ascariasis, trichuriasis, hookworm, and strongyloidiasis), LF, and scabies. We reviewed the literature to retrieve field studies examining the effect of ivermectin treatment on off-target infections and formulated assumptions about the impact of ivermectin mass treatment on the associated burden of disease. Next, we retrieved estimates of the disease burden of candidate off-target diseases from the Global Burden of Disease (GBD) 2010 Study [14]. By combining this information with data on the number of ivermectin treatments given through 2010 (recorded by APOC), we roughly estimated the number of DALYs due to off-target infections averted by APOC.

## Methods

### Assumptions about effect of ivermectin on burden of off-target infections

We first performed a systematic PubMed search to determine efficacy of ivermectin mono-treatment against off-target infection, defined as the ability to provide a clinically measurable and preferably beneficial effect. We used the key term “ivermectin” in combination with any of the following: “efficacy”, “mass treatment”, “morbidity control”. Searches were made without time limitations. If available, meta-analysis studies evaluating the efficacy of ivermectin against a specific disease were used. If meta-analysis studies were not available, clinical studies reporting the efficacy of treatment were selected if: (1) the treatment regime concerned a single dose of about 150–200 μg/kg of body weight, and (2) the efficacy was evaluated up to one month for STH infections and up to a year for filarial infections and EPSDs. We considered a month to be the threshold duration of the immediate effect of ivermectin on STH infections. For LF and EPSDs, longer periods were considered due to a lack of studies evaluating the efficacy of ivermectin as soon as one month after administration. Only studies reporting their results in terms of the following criteria were considered: (1) percent of patients cured and/or percent egg reduction for STH infections, (2) percent microfilaria (mf) reduction (microfilaricidal efficacy) and/or the percent reduction in female fecundity (embryostatic efficacy) in LF, and (3) percent of patients cured for EPSDs. For some EPSDs, clinical studies describing single cases were considered due to the rareness of their incidence. If repeated doses were given, it was noted.

Based on the results of the literature review, we formulated assumptions about the effect of ivermectin mono-treatment on the burden of (untreated) off-target infections, in terms of reduction in DALYs lost (Box 1). Assumptions were formulated while considering the following factors: the direct effect of ivermectin treatment on infection levels in individuals, the clinical manifestations of each disease, the short and long term effects of mass treatment on incidence and prevalence of morbidity, and the patterns of post-treatment re-infection. The effect of ivermectin was expressed as parameter *β*_*x*_ (range 0–1), which represents the average reduction in the burden of disease *x* in DALYs lost over a period of six years achieved thanks to mass treatment with ivermectin. The six-year period was based on APOC data on population coverage of ivermectin mass treatment, which suggest that most of the population in APOC areas has been subject to at least six rounds of mass treatment between 1995 and 2010 [3]. For infections such as STH and scabies, in which morbidity is highly correlated with intensity of infection (parasite load), and treatment only influences transmission to a small extent, we assumed that the impact of treatment is the same each year. For LF, repeated mass treatment rounds are expected to have an increasingly higher impact on the disease burden, through the effects of mass treatment on transmission and prevention of further exposure to infection that would lead to chronic disability (e.g. lymphedema). We assumed that for LF, parameter *β*_*x*_ represents the average health impact in all areas subject to varying periods of ivermectin mass treatment.

**Box 1. Reasoning and Assumptions Regarding the Effectiveness of an Average Round of Mass Treatment (Parameter *β*_*x*_)**

**Ascariasis**

A single dose of ivermectin is highly efficacious against *Ascaris lumbricoides*, reducing fecal egg counts by 94–100% [28,29], and clearing infection in 78–100% [28–32]. The clinical manifestations of ascariasis include: malnutrition, intestinal obstruction, growth and cognitive delays [15,33]. They are associated with high intensity of infection (worm burden) [34]. The immediate post-treatment health benefits include: weight/height gain, increased fitness and physical activity [7,35]. The long-term health benefits of treatment include: prevention of intestinal obstruction, increased school attendance, learning abilities, and cognitive testing [7,18,35,36]. Field studies show that post-treatment reinfection does not bring the worm burden or prevalence back to pre-treatment levels in treated communities [19,37,38]. Based on this information, we assume that an average round of mass treatment with ivermectin reduces the burden of ascariasis by 50% (*β*_*asc*_ = 0.5).

**Trichuriasis**

A single dose of ivermectin has medium to high efficacy against *Trichuris trichiura*, reducing fecal egg counts by 86–93% [29,30], and clearing infection in 35–67%[29–31]. The clinical manifestations of trichuriasis include: inflammatory bowel disease, growth and cognitive delays [15,33], which are associated with high intensity of infection (worm burden). The immediate post-treatment health benefits include: weight/height gain, increased fitness and physical activity [7,35]. The long-term health benefits of treatment include: prevention of inflammatory bowel disease, increased school attendance, learning abilities, and cognitive testing [7,18,35,36]. Field studies show that post-treatment reinfection does not bring the worm burden or prevalence back to pre-treatment levels in treated communities with high initial prevalence [19,36,37]. Based on this information, we assume that an average round of mass treatment with ivermectin reduces the burden of trichuriasis by 50% (*β*_*trich*_ = 0.5).

**Hookworm infections**

A single dose of ivermectin has low efficacy against *Ancylostoma duodenale* and *Necator americanus*, reducing fecal egg counts by 52–80% [29,31], and clearing infection in 12–33% [29,31,39]. The clinical manifestations of hookworm include: iron-deficiency anemia, malnutrition, growth and cognitive delays, and poor pregnancy outcomes [15,33]. They are associated with high intensity of infection (worm burden). A round of mass treatment has a small impact on the worm burden due to the low efficacy of the drug [37]. The immediate post-treatment health benefits include: weight/height gain, increased fitness [7,35]. The long-term health benefits of treatment include: prevention of anemia, malnutrition, growth and cognitive delays. Field studies show that post-treatment reinfection brings the prevalence back to pre-treatment values [19,36]. Based on this information, we assume that an average round of mass treatment with ivermectin reduces the burden of hookworm infections by 20% (*β*_*hook*_ = 0.2).

**Strongyloidiasis**

A single dose of ivermectin is highly efficacious against *Strongyloides stercoralis*, reducing fecal larval counts by 94–100% [29,30], and clearing infection in 83–100% [16,30,40–42]. Ivermectin is considered to be the drug of choice for treating strongyloidiasis [42–46]. A round of mass treatment rapidly lowers the worm burden the population [30,37,41,43]. The clinical manifestations of strongyloidiasis include: abdominal pain and discomfort, diarrhea, weight loss, pruritus, and the potentially deadly dissemination (hyperinfection) [45]. The immediate post-treatment health benefits include: prevention of abdominal pain and discomfort, prevention of diarrhea, weight gain [45,46]. The long-term health benefits of treatment include: prevention of potentially fatal dissemination of infection [46]. Although *S*. *stercoralis* can persist in an untreated individual for years due to autoinfection [47], once eradicated during treatment, an individual can only be re-infected from the environment. Since no follow up studies were found in the literature for *S*. *stercoralis* reinfection, we assume that the environmental reinfection rate would be similar to that of other STH infections. Based on this information, we assume that an average round of mass treatment with ivermectin reduces the morbidity due to strongyloidiasis by 50% (*β*_*strong*_ = 0.5).

**Lymphatic filariasis (LF)**

A single dose of ivermectin has high microfilaricidal (100%) and intermediate embryostatic (35%) efficacy against LF [48]. Microfilariae start to reappear at three months post treatment and reaches approximately 11% of pre-treatment values at twelve months [49]. The clinical manifestations of LF include: adenolymphangitis, lymphedema, and hydrocele. Existing morbidity is not cured or improved by clearing the microfilaria or even the complete and persistent clearance of infection. Yet, through their impact on transmission, repeated rounds of ivermectin mass treatment help to prevent the onset of new cases and possibly the progression of early clinical manifestations [50]. Annual ivermectin mass treatment does not interrupt the transmission of LF [51]. However, given population turnover and a reduced incidence (and possibly progression) of clinical manifestations, mass treatment indirectly decreases the prevalence of clinical symptoms of LF over time. Based on this information, we assume that an average round of mass treatment with ivermectin reduces the burden due to LF by 10% (*β*_*LF*_ = 0.1).

**Epidermal parasitic skin diseases (EPSDs)**

A single dose of ivermectin is highly efficacious against EPSDs [6], causing an immediate lowering of the intensity of infestation. One dose of ivermectin suppresses scabies infection for up to three months [52], and even clears infestation in 70–100% [53–55]. A round of mass treatment lowers the intensity of infestation rapidly [56,57]. The clinical manifestations of EPSDs include: pruritus (itching), and secondary streptococcal infections [9]. The immediate post-treatment health benefits include: prevention of secondary infections, decreased physical and mental discomfort of severe pruritus, increased libido [18,58,59]. The long-term health benefits of treatment include: prevention of streptococcal pyoderma which in turn predisposes to rheumatic fever, acute glomerulonephritis and their respective long-term sequelae: rheumatic heart disease and chronic renal insufficiency [9,60–63]. Based on this information, we assume that an average round of mass treatment with ivermectin reduces the morbidity due to EPSDs by 50% (*β*_*oncho*_ = 0.5)

### Data sources for estimates of disease burden

From the GBD 2010 study [14], we derived country-specific estimates of the burden per capita (DALYs lost per 100,000 persons) for ascariasis, trichuriasis, hookworm, LF, and EPSDs (scabies only) in 1995, 2000, 2005, and 2010 (extracted from the online GBD data visualization tool [20]). For the years in between, we assumed that the disease burden of these off-target infections followed a trend consistent with exponential interpolation of the available estimates. For some countries covered by APOC, the GBD 2010 study reports a decline in the burden of off-target diseases between 1995 and 2010. In the current study, we assume that this decline is not due to APOC or other control activities, at worst leading to an underestimation of the health impact of ivermectin mass treatment on off-target diseases (i.e. because the counterfactual burden without these activities would be even higher than reported by GBD 2010).

Though the group of EPSDs consists of several infections such as scabies, tungiasis (sand fleas), pediculosis (lice), and several other infections [9], for the purpose of this study, we only considered scabies, as burden estimates have been made only for this particular infection so far. Further, the GBD 2010 study does not provide estimates for the burden of strongyloidiasis, an STH, even though its prevalence in SSA is probably considerable (although of unknown size). We assumed that the burden due to strongyloidiasis amounts to 1/5 of the total burden caused by the three major STH infections (ascariasis, trichuriasis, and hookworm). This assumption was based on the estimate that the prevalence of the three major STH infections in SSA ranges between 20–50% [12], and a large cross-sectional study in rural Ghana which reported a prevalence of strongyloidiasis of 11.6% [21]. These figures suggest that the prevalence and presumably the burden of strongyloidiasis may be up to five times lower than the prevalence and burden of the other three more common STH infections. Given the uncertainty regarding the assumption, we varied it in the sensitivity analysis (details below).

### Calculation of disease burden averted

We calculated the disease burden averted, *Ma*_*iyx*_ (in DALYs), for each selected off-target infection *x* and summed it over the sixteen countries in which APOC has been active between 1995 and 2010 using the following formula, where *i* represents a specific APOC country and *y* represents the year of mass treatment:

$${Ma}_{x}=\sum_{i=1}^{16}\sum_{y=1995}^{2010}{Ma}_{iyx}=\sum_{i=1}^{16}\sum_{y=1995}^{2010}\beta_{x}T_{iy}h_{ix}\frac{1-pMc_{x}-pMw_{x}}{1-pc-pw}M_{iyx}$$

In this formula, *M*_*ixy*_ reflects the annual burden per capita due to infection *x* (as reported by the GBD 2010 study). This figure was adjusted to represent the burden per capita in population eligible for mass treatment with ivermectin in APOC areas by adjusting for over- or underrepresentation of the disease burden in children under five and pregnant or lactating women who are not eligible for ivermectin ($\frac{1-pMc_{x}-pMw_{x}}{1-pc-pw}$ is the fraction $\frac{\text{proportion of burden in ivermectin-eligible population}}{\text{proportion of population eligible for ivermectin}}$, where *pMc*_*x*_ + *pMw*_*x*_ is the proportion of the disease burden in children under five, and *pc* + *pw* is the proportion of children under five and pregnant or lactating women in the population). We further adjusted for clustering of disease burden in APOC regions compared to other country regions (*h*_*ix*_). The adjusted burden per capita ($h_{ix}\frac{1-pMc_{x}-pMw_{x}}{1-pc-pw}M_{ixy}$) was then multiplied by the number of treated individuals *T*_*iy*_ for any given year *y* (extracted from APOC records), yielding the potential disease burden in treated people. Assumptions about the values of aforementioned parameters can be found in Table 1, along with the associated literature references [3,11,12,14,20,22–27]. The potential disease burden in treated people was multiplied with the infection-specific ivermectin efficacy *β*_*x*_, yielding the estimated averted disease burden related to infection *x* in country *i* for year *y*. Results were then summed over years and countries, resulting in a total estimated number of DALYs averted (*Ma*_*x*_), related to infection *x*.

**Table 1:** Description of parameters and their values.

Parameter	Description	Assumption or reference
*M*_*ixy*_	Estimated burden of disease *x* per capita in country *i* and year *y* (1995–2010)	Global Burden of Disease 2010 study [14,20].
*T*_*iy*_	Number of people treated in country *i* and year *y* (1995–2010)	Coverage data from APOC records that were also used in a recent evaluation of the health impact and cost of APOC activities [3,4].
*h*_*ix*_	Heterogeneity index for disease *x* in country *i*	The spread of STH infections in APOC countries was assessed by visually comparing REMO maps with maps published by the Global Atlas of Helminth Infections [12,25]. *h* = 1.0, when STH infections are highly prevalent across the whole country and 1.5 when certain areas of high prevalence of STH infections overlap APOC regions. For LF, *h* = 1.0 for all countries. For all other infections, *h* = 1.5 for CAR, Chad and Nigeria (as APOC covers about half of the aforementioned countries, the hypothetical maximum value of *h* would be 2.0 for those countries). For all other countries, we assumed *h* equal to 1.0.
*pc* + *pw*	Proportion of children below five years of age and pregnant women in the population	Assumed to be 0.2 for all countries, based on data from U.S. Census Bureau [27].
*pMc*_*x*_ + *pMw*_*x*_	Proportion of disease burden in pregnant women and children under the age of five years	*pMc*_*x*_ + *pMw*_*x*_ = 0.2 if burden is evenly distributed over pregnant women, children under five, and the rest of the population. For ascariasis and trichuriasis, *pMc*_*x*_ + *pMw*_*x*_ = 0.1, since the burden in young children is considered substantial [23,24], though highest in those eligible for treatment (5–14 years) [14]. For hookworm, *pMc*_*x*_ + *pMw*_*x*_ = 0.3, since the burden in pregnant women is considered to be substantial [22,26].
*β*_*x*_	The average annual reduction in DALYs due to infection *x* over six annual rounds of treatment	See Box 1.

### Sensitivity analysis

A univariate sensitivity analysis was performed to assess how the main result (number of DALYs averted related to off-target infections) changed when parameter *β*_*x*_ was increased or decreased by 20%. The value of 20% was chosen because larger increases or decreases of the effectiveness of ivermectin against off-target infections are highly unlikely to occur in field settings. Likewise, the assigned value of *h*_*ix*_ was varied by ±20% for those countries where *h*_*ix*_ was initially set to 1.5 (see Table 1). In addition, prevalence and burden of STH infections or EPSDs were assumed to either be overrepresented in APOC regions for all or none of the countries covered by APOC. The impact of the assumption regarding burden due to strongyloidiasis (1/5 of the total burden due to other STH infections) was examined by halving or doubling the assumed proportion. Finally, we also performed a multivariate sensitivity analysis by simultaneously increasing or decreasing all assumed *β*_*x*_ parameters by 20% as an extreme scenario.

## Results

We assumed that each year, mass treatment would avert some fraction *β*_*x*_ of the potential disease burden in treated communities. Based on literature, this fraction was assumed to be 0.5 for ascariasis, trichuriasis, strongyloidiasis and EPSDs, 0.2 for hookworm infections, and 0.1 for LF (Box 1).

We estimated that without APOC (counterfactual situation assuming that there is no mass treatment with ivermectin by APOC), the potential disease burden of STH infections, lymphatic filariasis, and EPSD in individuals otherwise treated with ivermectin would amount to a cumulative burden of 1.7 million DALYs between 1995 and 2010. Of these, 493 thousand DALYs were averted by APOC through ivermectin mass treatment (Table 2). Most of the DALYs averted by APOC were related to ascariasis (162 thousand) and scabies (116 thousand), followed by LF (71 thousand), strongyloidiasis (67 thousand), and hookworm infection (61 thousand). Only a small part of the burden averted by APOC was related to trichuriasis (17 thousand). Nigeria contributed 53% of the total averted number of DALYs related to off-target infections (260 thousand of 493 thousand), followed by the DRC (62 thousand or 13%) and Cameroon (59 thousand or 12%).

**Table 2:** Burden of off-target infections averted by annual ivermectin mass treatment with ivermectin in Africa. Figures represent the cumulative burden averted between 1995 and 2010 in areas covered by the African Programme for Onchocerciasis Control.

	Burden averted by ivermectin mass treatment (DALYs x 1,000)^a^							
Country	Ascariasis	Trichur-iasis	Hook-worm	Strongy-loidiasis	Lymphatic filariasis	Scabies	Total	(%)
Angola	0.2	0.1	0.2	0.1	0.2	0.2	**0.9**	(0.2%)
Burundi	0.4	0.1	1.3	0.7	0.8	1.2	**4.6**	(0.9%)
Cameroon	23.6	6.1	5.7	7.2	8.5	8.0	**59.3**	(12%)
Central African Republic	0.5	0.2	4.3	0.3	2.6	3.0	**10.9**	(2.2%)
Chad	0.1	0.0	3.2	2.1	1.8	8.6	**15.8**	(3.2%)
Congo	1.5	0.8	0.6	0.5	0.8	0.8	**5.1**	(1%)
Democratic Republic of Congo	18.1	5.5	11.9	1.6	11.0	14.0	**62.0**	(12.6%)
Equatorial Guinea	0.1	0.1	0.0	0.0	0.1	0.0	**0.3**	(0.1%)
Ethiopia	2.6	1.7	2.5	0.1	2.2	8.0	**17.2**	(3.5%)
Liberia	1.1	0.5	3.0	3.7	2.0	2.9	**13.2**	(2.7%)
Malawi	0.3	0.0	0.7	2.6	0.4	5.3	**9.2**	(1.9%)
Nigeria	112.0	1.2	20.5	45.3	31.8	49.1	**259.9**	(52.8%)
Sudan and South Sudan^b^	0.1	0.0	0.9	1.9	0.6	5.5	**9.0**	(1.8%)
Uganda	0.6	0.2	3.3	3.4	2.0	4.2	**13.8**	(2.8%)
United Republic of Tanzania	0.2	0.5	2.9	1.2	1.8	4.9	**11.5**	(2.3%)
Total	161.5	16.9	61.0	70.9	66.6	115.7	**492.5**	(100%)

^a^ These figures are the product of the potential disease burden due to off-target infections in people treated with ivermectin and the assumed effect of ivermectin treatment on the disease burden (see Box 1).

^b^ Estimates for Sudan and South Sudan are merged, as information on the burden per capita was reported for the two together [14].

**Fig 1:**
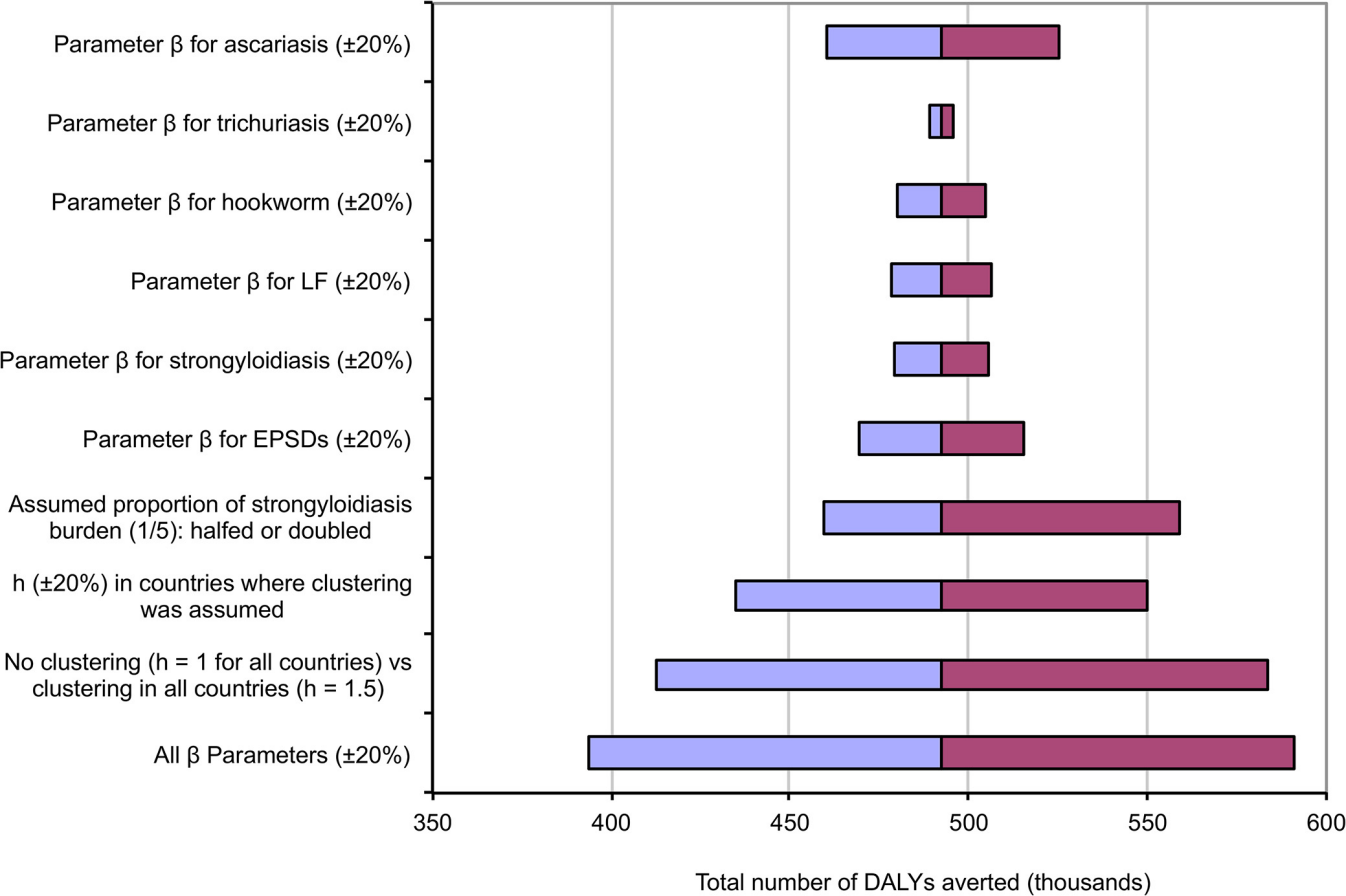
Sensitivity analysis for the impact of assumed parameter values on the total averted burden. For each parameter (y-axis), the figure between brackets indicates the relative amount by which it was varied in the sensitivity analysis.

Fig 1 shows the results of the sensitivity analyses. Changing individual *β*_*x*_ parameters by 20% resulted in estimates very similar to the main estimate. Obviously, increasing or decreasing all *β*_*x*_ parameters simultaneously by 20% resulted in ±20% deviations from the main estimate of 493 thousand DALYs averted. Assumed no clustering of STH and EPSDs in APOC areas (*h*_*ix*_ = 1.0) or clustering of all infections but LF in all countries (*h*_*ix*_ = 1.5) resulted in 16% lower and 19% higher estimates of total DALYs averted, respectively.

## Discussion

The impact of APOC on off-target NTDs has previously been discussed and considered to be important, but difficult to quantify. We estimated that if APOC would not have been there, STH infections, strongyloidiasis, and scabies would have caused a cumulative burden of 1.7 million DALYs lost between 1995 and 2010 in individuals who would otherwise have been treated with ivermectin. We roughly estimated that of these 1.7 million DALYs, mass treatment with ivermectin has averted 500 thousand DALYs. This means that apart from the impact of APOC on the burden of onchocerciasis (8.9 million DALYs averted), there has been an additional 5.5% health impact through the effect of ivermectin mass treatment on off-target NTDs. This indicates that the cost-effectiveness of APOC is even somewhat higher than previously estimated.

The estimate of 500 thousand additionally averted DALYs was based on a simple approach that included assumptions about the impact of mass treatment on the burden of selected off-target infections endemic in APOC countries. Because we considered a period of six years for estimating the effects of ivermectin mass treatment (i.e. the minimum duration of most APOC programs), our approach may underestimate the averted burden in countries where ivermectin mass treatments have taken place for over six years (i.e. where effects on transmission may be larger). Also, we did not take into account the protective impact of ivermectin mass treatment due to a reduced transmission to children under five years of age and pregnant women (who receive no ivermectin). Gutman *et al*. show that the prevalence of some STH infections was significantly lower in pre-school children living in treated communities compared to pre-school children living in non-treated communities [19]. Further, we assumed that APOC interventions have not been accounted for in the burden estimates for off-target NTDs provided by the GBD 2010 study, meaning that at worst (if GBD 2010 does account for APOC), ivermectin mass treatment has had a larger health impact than we estimate here. Also, ivermectin mass treatment probably has an effect on the burden of relatively rare or minor infections that were excluded from our analysis, such as enterobiasis, loiasis, streptocerciasis, serous cavity filariasis, and EPSDs other than scabies. Furthermore, ivermectin mass treatment also has a–yet to be quantified–effect on malaria transmission through the endectocidal effects of ivermectin on *Anopheles* vectors [64]. On the other hand, we did not consider the burden of severe adverse effects of ivermectin treatment related to loiasis [65,66], which is endemic in parts of the APOC region [67]. Overall, if anything, our results underestimate the true impact of APOC activities on off-target infections.

Our estimates of the impact of APOC on the burden of off-target diseases could be further refined with more sophisticated approaches, such as mathematical modeling. For some of the off-target infections, mathematical models have already been developed, such as for transmission and morbidity due ascariasis and transmission of lymphatic filariasis [22,68]. Epidemiological data and understanding of the mechanisms through which parasitic infections cause morbidity in the human host are needed to develop similar models for other parasitic infection, and update currently existing models. However, estimates made with such models are only usefully accurate if they are based on good information about the distribution of worms in host populations. Since such data are not yet widely available and the development of mathematical models is time-consuming and expensive, obtaining more precise estimates of the (averted) burden of off-target infections remains a challenge.

We ignored that in 16 countries, ivermectin mass treatment was planned to be combined with albendazole to target both onchocerciasis and LF from 2000 onwards [8]. However, 11 countries had not provided any LF treatment by 2010 (Angola, Burundi, Chad, Congo, DRC, Equatorial Guinea, Liberia, Sudan, South Sudan), and two countries had only implemented 1–2 treatment rounds reaching <6% of the target population (Ethiopia and Central African Republic) [69]. Only five countries (Cameroon, Malawi, Nigeria, Uganda, United Republic of Tanzania) had more mature LF elimination programmes that supplied 3–11 treatment rounds through 2010, reaching 25%–83% of the LF target population. We lack information on whether LF treatments took place in areas with LF only or co-endemic areas, but countries might have started in areas where mass treatment already took place for onchocerciasis. For these countries (especially Nigeria where many people live in co-endemic areas) it is therefore perhaps not justified to fully attribute the estimated effects on off-target diseases to APOC alone. It would be more useful to estimate the overall effect of repeated mass treatments on all target and off-target diseases; such estimates would be larger than the figures presented here, thanks to the addition of albendazole.

The so-called neglected tropical diseases (consisting of STH, filariases, EPSDs, and several other infections) are the most common conditions affecting the poorest 500 million people living in SSA [10,15]. The infections covered in our analysis have been estimated to be responsible for a burden of 8.3 million DALYs lost in SSA in 2010 [14]. Compared to this, our estimate of the health impact of ivermectin mass treatment on off-target diseases is modest (493 thousand DALYs averted). In order to enhance the impact of mass treatment on LF and STH infections, it would be interesting to consider adding albendazole or other STH-specific drugs to mass treatments (targeting the appropriate age-groups). For instance, in a counterfactual scenario where APOC would have added albendazole to their community-directed treatment strategy from 2000 onwards, an additional 389 thousand DALYs would have been averted by 2010, assuming that MDA would have had a higher impact on ascariasis, trichuriasis, hookworm, and LF (i.e. *β*_*STH*_ = 0.9, instead of 0.5; *β*_*LF*_ = 0.3, instead of 0.1). Guidelines for what anthelminthic drugs should be used in different areas have already been formulated [70]. Further, to sustain the off-target health impact after onchocerciasis and LF have been eliminated from APOC target areas, it should be considered to continue mass treatments against STH with albendazole (possibly combined with ivermectin against trichuriasis [71]), either school-based (mainly covering the burden of ascariasis and trichuriasis in children) or community-based (covering the burden of all STH). This would also be in line with the London Declaration on Neglected Tropical Diseases [72,73].

In conclusion, we roughly estimated that ivermectin mass treatment coordinated by APOC has averted about 500 thousand DALYs related to off-target infections. This health impact constitutes an additional 5.5% on top of the impact of APOC on the burden of onchocerciasis, and indicates that the cost-effectiveness of APOC is even higher than previously estimated. To amplify and sustain this additional health impact, control programs could consider adding albendazole to mass treatments, and continue this after elimination of onchocerciasis and lymphatic filariasis.

## Supporting Information

S1 File Calculations and input data. Excel spreadsheet.
(XLS)
